# The effectiveness and safety of acupuncture for patients with myopia: A protocol for a systematic review and meta-analysis

**DOI:** 10.1097/MD.0000000000020410

**Published:** 2020-06-05

**Authors:** Zengfang Yu, Xinxin Wang, Xiaoshuang Zhao, Dan Li, Junguo Duan

**Affiliations:** Eye School, Chengdu University of Traditional Chinese Medicine, Chengdu, Sichuan, China.

**Keywords:** acupuncture, children and adolescent, meta-analysis protocol, myopia

## Abstract

**Background:**

Myopia is a common visual disorder which has become a public health problem worldwide. Myopia and high myopia are substantial risk factors for severe visual impairment and other serious eye diseases. Acupuncture used to prevent and control myopia is a common practice in China, but it is controversial in other countries. This study aims to evaluate the efficacy and safety of acupuncture in delaying the progression of myopia in children and adolescents through systematic evaluation.

**Methods and analysis:**

The following electronic databases will be searched from inception to November 2019 regardless of publication status and language: Medline, EMBASE, Web of Science, the Cochrane Library, PubMed, China National Knowledge Infrastructure (CNKI), China Biology Medicine (CBM), Chinese Scientific Journal Database (VIP), Wanfang Database, Chinese Biomedical Literature Database (CBLD), Chinese Science and Technology Periodical Database (CSTPD). RCT registration websites, including http://www.ClinicalTrials.gov and http://www.chictr.org.cn, will also be searched. Review Manager V.5.3 will be used to analysis the statistic. Two reviewers (ZY and XW) will independently select studies, extract and code the data, assess risk of bias of the included studies, evaluate the quality of evidence for outcomes.

**Results:**

This study will provide a rational synthesis of current evidences for acupuncture to delay the progression for myopia in children and adolescents.

**Conclusion:**

The conclusion of this study will provide evidence for evaluating the efficacy and safety of acupuncture to delay the progression for myopia in children and adolescents.

## Abstract 2

Supplemental Digital Content is available in the text

## 1 Introduction

Acupuncture and related treatments have shown clinical effects for myopia in children and adolescents in many studies. To our knowledge, most trials on acupuncture for myopia in children and adolescents have small sample size. There is a lack of high-quality evidence on acupuncture beneficial to myopia in children and adolescents. Thus, this systematic review aims to assess the efficacy and safety of acupuncture to delay in progression for myopia in children and adolescents.

### 1.1 Description of the condition

Myopia is a common cause of vision loss and has become a relatively prevalent and increasing public health concern in the world, especially in East Asia.^[1]^ It is generally defined as a spherical refractive error caused by excessive refractive power and/or axial lengthening of the eye, which result in anterior displacement of focus from the retina. The prevalence of myopia, especially high myopia, has increased significantly over the past half century. A research predicted that there will be 49.8% of the world population with myopia (4758 million people) and 9.8% of them with high myopia (938 million people) by 2050.^[2]^ From 1985 to 2010, the prevalence of myopia among primary and secondary school students in China increased from 28.6% to 56.8%, and the number of myopia will increase to 152.4 million in 2020 and 180.4 million in 2030.^[3]^ It has been estimated that prevalence of myopia in children and adolescents ages 3 to 19 is about 84% by 2050 in China.^[4]^ Especially high myopia can be complicated by a number of vision-compromising conditions such as retinal detachment, primary open angle glaucoma, cataract and macular degeneration. Myopia has become one of the main causes of irreversible blindness and visual impairment.^[5–6]^ Myopia is associated with urban living environments, emphasis on education, outdoor activities and ethnic differences.^[7]^

### 1.2 Description of the intervention

Myopia is one of the most common eye problems in children and adolescents. It usually occurs around the age of 6 to 8, progresses between the ages of 13 and 16, and stabilizes at the age of 16^[8]^ or into early adulthood. Early detection and treatment of myopia is associated with better vision improvement and correction. Nowadays, a number of optical and pharmacological modalities have been widely investigated for restriction of myopia progression, such as wearing glasses and/or contact lenses or using atropine eyedrops. But wearing glasses can’t control the progression of myopia, while atropine has not been approved by the Food and Drug Administration (FDA). Acupuncture, a major component of traditional Chinese medicine, has been extensively used by clinicians for the management and prophylaxis of myopia for long time. In 1980, the World Health Organization (WHO) recommended acupuncture as an effective alternative therapy. Previous studies have shown that acupuncture appears to have a positive effect on slowing the progression of myopia.^[9–17]^ Therefore, acupuncture has the potential to be an effective treatment option to delay the progression for myopia.

### 1.3 Why it is important to this review

However, the effect of acupuncture for myopia is still controversial from the perspective of evidence-based medicine. One systematic review has been conducted to assess the clinical benefits of acupuncture therapy for myopia, but this study which only reviewed two studies before 2011 without meta-analysis.^[18]^ However, at present there exists no updated systematic review or study protocol published on this question. We thus have a unique opportunity to re-evaluate the issue and conceive this systematic review to determine the effectiveness and safety of acupuncture for patients with myopia based on the most comprehensive and up-to-date resources.

### 1.4 Objectives

This study aims to evaluate the efficacy and safety of acupuncture in delaying the progression of myopia in children and adolescents through systematic evaluation.

## 2 Methods

### 2.1 Study registration

PROSPERO systematical review protocol registration number is CRD42020149152. This protocol will base on the preferred reporting items for systematic reviews and meta-analyses protocols (PRISMA-P) statement guidelines.^[19]^

### 2.2 Criteria for considering studies for this review

#### 2.2.1 Type of studies

Only randomized controlled trials (RCTs) of acupuncture therapy for Myopia will be included in this review. Quasi-RCTs, cluster RCTs, non-randomized clinical studies, observational studies, case studies and experimental studies will be excluded. Dissertations and abstracts will be included if these studies contain sufficient details for critical evaluation. There will be no restriction regarding publication and language that the studies are published in and only published studies will be included.

#### 2.2.2 Type of participants

Articles involving participants are children and adolescents aged 18 or younger (at baseline), diagnosed with myopia which defined as spherical equivalent refraction ≤ −6.00 diopters, and cylindrical lenses <1.5 D, and without any ocular comorbidities including strabismus and amblyopia.

Articles involving participants with Less than −0.5 diopters; Any participants with ocular pathology by external and internal eye examination; cardiologic, neurologic, nephrologic, hepatologic, hematologic, or psychiatric disorders; chronic diseases requiring medication that must not be interrupted will be excluded. Studies related to surgical interventions for myopia correction, for example, refractive surgery will not be considered.

#### 2.2.3 Type of interventions and controls

The interventions including acupuncture, warm-acupuncture, electroacupuncture and so on, only acupuncture therapies and combination of several acupuncture approaches will be included. Studies combined acupuncture with other therapies will be excluded. No restriction on duration and dose of treatment, if applicable, will be imposed.

Sham-acupuncture (sham acupuncture is placebo acupuncture in which needles are inserted in the skin but are not intended to stimulate known acupuncture points),^[20–23]^ no intervention, placebo/ sham therapy, medicine, non-specific treatment(such as vitamin E) and glass will be permissible as the comparator.

#### 2.2.4 Type of outcome measures

##### 2.2.4.1 Primary outcomes

Myopia progression and axial elongation will be evaluated as the primary outcome. Myopia progression was assessed as an average change in refractive error, measured in diopters.^[24]^ Average change in axial length,^[25]^ measured in millimeters, was also evaluated.

##### 2.2.4.2 Secondary outcomes

Diopter and distance visual acuity^[26]^ will be evaluated as the secondary outcomes.

### 2.3 Search methods for identifying the studies

#### 2.3.1 Electronic searches

The following electronic databases will be searched from inception to November 2019 regardless of publication status and language: Medline, EMBASE, Web of Science, the Cochrane Library, PubMed, CNKI, CBM, VIP, Wanfang Database, CBLD, CSTPD. RCT registration websites, including http://www.ClinicalTrials.gov and http://www.chictr.org.cn, will also be searched.

#### 2.3.2 Searches of other resources

In addition, the references in all the located articles and the references lists of identified publications, comments, reviews and overviews will also be manually searched for further relevant articles. Dissertations and abstracts will be included. Ambiguous literature will be investigated manually to avoid missing eligible trials. The WHO International Clinical Trials Registry Platform, Chinese Clinical Trial Registry, Google Scholar and ClinicalTrials.gov will also be searched to identify any planned, ongoing or unpublished literature.

#### 2.3.3 Study strategies

The search strategy can be seen in the online supplementary appendix.

The validity of the search strategy was demonstrated by the ability to identify recent qualitative reviews of myopia.^[27–29]^

### 2.4 Data collection and analysis

#### 2.4.1 Selection of studies

**Figure 1:**
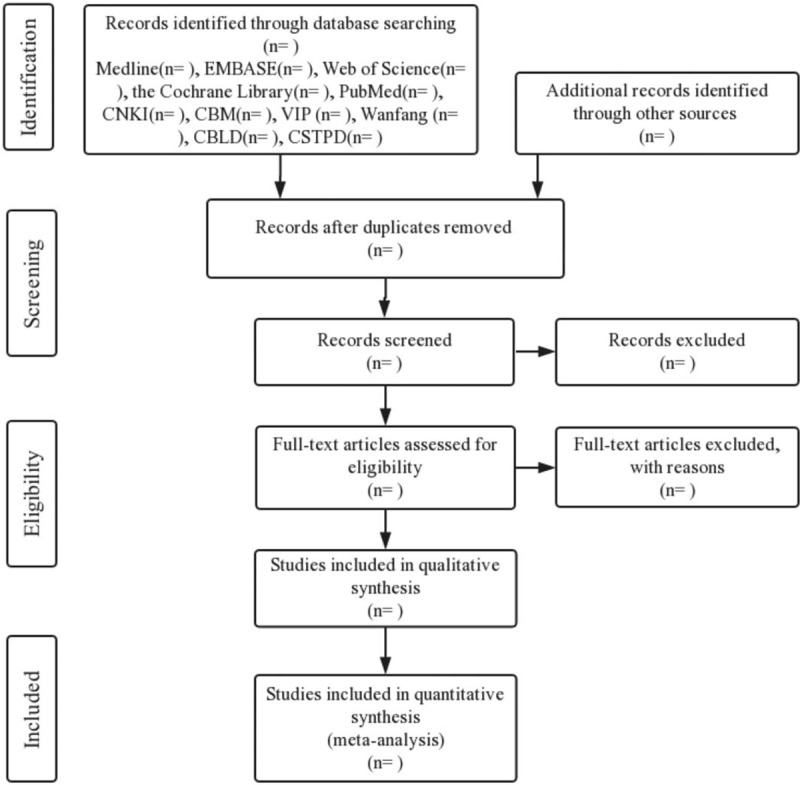
Flow diagram of study selection process.

All the retrieved studies will be imported into an EndNote library and duplicate studies will be deleted. Two reviewers (ZY and XW) will independently screen the titles and abstracts of the retrieved studies in accordance with the inclusion and exclusion criteria and crosscheck. The disagreement will be resolved by consensus or consultation with a third independent researcher (XZ). The details of the selection process will be illustrated in preferred reporting items for systematic review and meta-analysis flow diagram (Fig. 1).

#### 2.4.2 Data extraction and management

Two reviewers (ZY and XW) will independently use a pre-designed standardized data extraction form to extract information from the included articles. The extract information includes general information (e.g., title, authors, year, and published country), details of study (e.g., design, inclusion and exclusion criteria, blinding, randomisation and sample size), participant characteristics (e.g., sex, age, number of subjects, axial elongation, Diopter and distance visual acuity), interventions (frequency, duration, study period, and so on), outcomes, adverse events and other detailed information. Details regarding the acupuncture and control interventions will be extracted on the basis of the revised standards for reporting interventions in clinical trials of acupuncture. If necessary, we will contact the corresponding authors of trials as much as possible for further information. Then the data acquired by two reviewers will be crosschecked to make sure there are no mistakes. If there are some differences, it will be resolved by team discussion.

### 2.5 Assessment of risk of bias in included studies

The two reviewers (ZY and XW) will independently use the Cochrane^[30]^ risk of bias tool to evaluated the qualities of the included trials based on six domains: random sequence generation, allocation concealment, blinding of participants and personnel, blinding of outcome assessment, selective reporting, incomplete outcome data and other bias. Blinding of practitioner is not possible because of the nature of acupuncture; therefore, only the blinding of participants and outcome assessors will be evaluated. The risk of bias for the individual domains will be classified as low, unclear (insufficient information provided) or high. Then the 2 researchers crosscheck to make sure there is no mistake. Disagreements will be resolved through discussion and consensus with a third researcher (XZ). Review Manager V.5.3 will be used to make the risk of bias diagram.

### 2.6 Assessment of the quality of acupuncture

The two reviewers (ZY and XW) will independently evaluate the quality of evidence for outcomes by using the method for grading of recommendations assessment, development and evaluation.^[31]^ The primary outcomes will be graded into ‘high’, ‘moderate’, ‘low’, or ‘very low’. Disagreements will be resolved through discussion and consensus with a third researcher (XZ).

### 2.7 Measures of treatment effect

For dichotomous data, the Risk ratios will be used with 95% CIs. Mean difference (MD) or standard MD will be used to measure the therapeutic effect for continuous data.

### 2.8 Dealing with missing data

If missing data are detected, we will attempt to contact the original study investigators by email or phone to acquire any missing or incomplete information if possible. If we cannot acquire accurate data through the above methods, we will exclude these studies.

### 2.9 Assessment of heterogeneity

*I*^*2*^ statistic and Q test (χ^2^) will be used for the assessment of heterogeneity which recommended by the Cochrane Handbook for Systematic Reviews of Interventions. If the *P* value in χ^2^ test is >.10 or *I*^*2*^ is <50%, the heterogeneity across studies is no obvious heterogeneity, a random effects model will be used. If the *P* value in χ^2^ test is <.10 or *I*^*2*^ is >50%, the heterogeneity across studies is statistically significant, a random effects model will be used. Moreover, a subgroup analysis or meta-regression will be conducted to explore the causes of heterogeneity among results of studies.

### 2.10 Assessment of reporting biases

The small-study will use the funnel plots to detect the reporting biases and effect. If more than 10 studies are included, the Egger method will be used to test the funnel plot asymmetry. If the funnel plots are found to be asymmetrical, we will try to interpret funnel plot asymmetry.

### 2.11 Data synthesis

The Review Manager V.5.3 (the Cochrane Collaboration) software will be conducted using to analysis all statistical. The meta-analysis will be used to combine trials with the same interventions and outcomes in similar populations to assess the effectiveness of the combined intervention. Dichotomous data will be expressed as relative risk and continuous variable as MD of 95% CI. If *I*^*2*^ < 50%, the fixed-effects model will be used for data synthesis. If *I*^*2*^ > 50%, we will use the random effects model to merge the data. When the meta-analysis is not applicable, we will be conducted describing descriptively the results.

### 2.12 Subgroup analysis and investigation of heterogeneity

If the necessary, subgroup analysis and sensitivity analysis will be performed to explore possible causes of heterogeneity. The subgroup analysis will be conducted based on the following topics: sex, diopter, progress classification, astigmatism, the type of acupuncture and control group, length of treatment, and duration, frequency. If quantitative synthesis is not applicable, we will proceed to narrative synthesis.

### 2.13 Sensitivity analysis

To identify the robustness of the study conclusions, the sensitivity analysis of primary outcomes will be employed according to following conditions: methodological quality, study quality, sample size, missing data, and analysis methods.

### 2.14 Ethics and dissemination

There is no necessity to acquire an ethical approval for this study, since there is no private information about the participants. The results of this study will be disseminated though a peer-reviewed journal or conference presentation. Important protocol revisions will be documented and updated on PROSPERO.

## 3 Discussion

First, in this study, our search strategy is comprehensive; Second, the two authors will independently conduct study selection, data extraction, and bias risk assessment. But, this study had some potential limitations. Different types of acupuncture may result in considerable heterogeneity. Subgroup analysis may address this problem and ensure consistency of interventions, but it may reduce the comparability of included studies and increase the difficulty of meta-analysis. We hope this study will lead to a better understanding of the effects of acupuncture on myopia in children and adolescents. If this agreement must be amended, we will provide an explanation of the change and the corresponding reason on the date of each amendment.

## Author contribution

Junguo Duan is the guarantor, Zengfang Yu, Xinxin Wang and Junguo Duan contributed to the conception of the study. The manuscript was drafted by Zengfang Yu and revised by Junguo Duan. The search strategy was developed by all authors and will be run by Zengfang Yu and Xinxin Wang. They will also independently screen the studies, extract data from included studies, assess the risk of bias and finish the data synthesis. The disagreement will be arbitrated by Xiaoshuang Zhao, who will ensure no errors occur during the study. All review authors have approved the final version of the protocol.

**Conceptualization:** Zengfang Yu, Xinxin Wang, junguo Duan.

**Data curation:** Xiaoshuang Zhao.

**Investigation:** Dan Li.

**Methodology:** Zengfang Yu, Xinxin Wang.

**Resources:** Dan Li.

**Software:** Zengfang Yu, Xinxin Wang, Xiaoshuang Zhao.

**Supervision:** junguo Duan.

**Writing – original draft:** Zengfang Yu.

**Writing – review & editing:** junguo Duan.

## Supplementary Material

Supplemental Digital Content
