# Supplementary material for: The effectiveness and safety of acupuncture for patients with myopia: A protocol for a systematic review and meta-analysis
Source: Medicine (Baltimore). 2020 Jun 5;99(23):e20410. doi: 10.1097/MD.0000000000020410 (PMC7306361; doi:10.1097/MD.0000000000020410)
Supplement: Supplemental Digital Content [file medi-99-e20410-s001.doc]

Relevant studies will be obtained from English database Medline, EMBASE, Web of Science, the Cochrane Library, PubMed; Chinese Database of China National Knowledge Infrastructure(CNKI), China Biology Medicine(CBM), Chinese Scientific Journal Database (VIP), Wanfang Database, Chinese Biomedical Literature Database, Chinese Science and Technology Periodical Database

Search Strategy for PubMed

#1 acupuncture [mh]

#2 Pharmacopuncture [all fields]

#3 acupuncture therapy [mh]

#4 Acupuncture Treatment [all fields]

#5 Pharmacoacupuncture Treatment [all fields]

#6 Pharmacoacupuncture Therapy [all fields]

#7 Acupotomy [all fields]

#8 Acupotomies [all fields]

#9 Ear acupuncture [mh]

#10 Auricular Acupuncture [all fields]

#11 Acupuncture point [mh]

#12 Acupoints [all fields]

#13 electroacupuncture [mh]

#14 dry needling [mh]

#15 Moxabustion [mh]

#16 scalp acupuncture [all fields]

#17 fire needling [all fields]

#18 Cutaneous acupuncture [all fields]

#19 elongated needle [all fields]

#20 intradermal needling [all fields]

#21 dermal needling [all fields]

#22 manual acupuncture [all fields]

#23 body acupuncture [all fields]

#24 warm-acupuncture [all fields]

#25 Wrist-Ankle acupuncture [all fields]

#26 abdominal acupuncture [all fields]

#27 #1 or #2 or #3 or #4 or #5 or #6 or #7 or #8 or #9 or #10 or #11 or #12 or #13 or #14 or #15 or #16 or #17 or #19 or #20 or #21 or #22 or #23 or #24 or #25 or #26

#28 myopias [mh]

#29 Nearsightedness [all fields]

#30 Nearsightednesses [all fields]

#31Short-sightedness [mh]

#32 near-sightedness [mh]

#33 visual health [all fields]

#34 refractive error [mh]

#35 Refractive Disorders [all fields]

#36 Ametropia [all fields]

#37 ocular refraction [mh]

#38 #28 or #29 or #30 or #31 or #32 or #33 or #34 or #35 or #36 or #37

#39 child [mh]

#40 Children [all fields]

#41 childhood [all fields]

#42 adolescent [mh]

#43 Adolescence [all fields]

#44 Teens [all fields]

#45 Teenagers [all fields]

#46 School-age [all fields]

#47 Schoolchildren [all fields]

#48 Childhood [all fields]

#49 Primary and secondary school students [all fields]

#50 #39 or #40 or #41 or #42 or #43 or #44 or #45 or #46 or #47 #48 or #49

#51 #38 and #50

#52 Randomized Controlled Trial [pt]

#53 Controlled Clinical Trial [pt]

#54 Trials [pt]

#55 Clinical Study [pt]

#56 Clinical Trial [pt]

#57 Randomized [pt]

#58 Randomized Clinical [pt]

#59 Pragmatic Clinical Trial [pt]

#60 #52 or #53 or #54 or #55 or #56 or #57 or #58 or #59

#61 #27 and #51 and #60
